# Financial toxicity profiles and their associations with healthcare utilization and catastrophic health expenditure in patients with primary liver cancer

**DOI:** 10.1097/MD.0000000000049816

**Published:** 2026-07-24

**Authors:** Hongwei Li, Lihong Yang, Li Zhao, Wan Wang, Hanxue Mei

**Affiliations:** a School of Nursing, Chifeng University, Chifeng, Inner Mongolia Autonomous Region, China; b The Second Ward of Neurology, Chifeng Hospital, Chifeng, Inner Mongolia Autonomous Region, China.

**Keywords:** catastrophic health expenditure, financial toxicity, healthcare utilization, hepatocellular carcinoma, latent profile analysis, out-of-pocket spending, primary liver cancer

## Abstract

Financial toxicity (FT) is increasingly recognized as an important patient-centered burden in cancer care. However, the heterogeneity of FT and its associations with healthcare utilization and catastrophic health expenditure (CHE) among patients with primary liver cancer (PLC) remain insufficiently understood. This study aimed to identify latent profiles of FT among patients with PLC and to examine their associations with healthcare utilization and CHE. We conducted a retrospective cohort study of consecutive adult patients with PLC who received inpatient and/or outpatient care between December 2024 and December 2025 (N = 720). FT was assessed using the COmprehensive Score for FT and multidimensional hardship indicators, including material hardship, financial worry, and coping behaviors. Latent profile analysis was used to identify FT profiles. Healthcare utilization outcomes included annual counts of outpatient visits, inpatient admissions, and emergency department visits. CHE was defined as annual out-of-pocket spending exceeding 40% of household capacity to pay, with a 25% threshold used in sensitivity analyses. Associations between FT profiles and utilization were estimated using covariate-adjusted negative binomial regression, and associations with CHE were estimated using covariate-adjusted logistic regression. A 3-profile solution was identified, comprising low FT (45.0%, n = 324), moderate FT (37.5%, n = 270), and high FT (17.5%, n = 126). Overall, the mean COmprehensive Score for FT score was 25.0 (standard deviation 10.9), and CHE occurred in 27.0% of participants. Compared with the low FT profile, the moderate and high FT profiles were associated with lower outpatient visit rates but higher inpatient admission and emergency department visit rates. CHE prevalence increased stepwise across the low, moderate, and high FT profiles. In adjusted models, moderate FT and high FT were associated with higher odds of CHE compared with low FT. Findings were robust using the 25% capacity-to-pay threshold. Patients with PLC exhibit distinct FT profiles that are strongly associated with healthcare utilization patterns and CHE risk. Routine FT screening and profile-informed interventions may help improve care continuity and strengthen financial protection in PLC management.

## 1. Introduction

Primary liver cancer (PLC), most commonly hepatocellular carcinoma (HCC), remains a major global health challenge with substantial mortality and an increasing share of cancer deaths. Global surveillance data from the World Health Organization (WHO) Global Cancer Observatory highlight the considerable incidence and mortality of liver cancer worldwide, underscoring the population-level burden and the need for comprehensive strategies that extend beyond survival to include patient-centered and health-system outcomes.^[1]^ In the present study, PLC was used as an umbrella term for primary malignant tumors of the liver. Because HCC represents the predominant subtype of PLC and much of the available economic-burden literature focuses on HCC, HCC-specific evidence is cited where relevant to contextualize the findings.

Alongside clinical complexity, PLC care often generates substantial economic consequences for patients and families. The management of HCC frequently involves intensive diagnostics, multimodal treatment (surgery, locoregional therapy, systemic therapy), and long-term surveillance, which can translate into meaningful out-of-pocket (OOP) spending and broader socioeconomic disruption. Recent empirical research in the United States has described measurable patient financial burden and OOP costs among individuals with HCC, emphasizing that economic strain is a practical and persistent dimension of care.^[2]^ Medicare-based evidence similarly suggests that HCC is associated with high healthcare costs and financial burden, reinforcing the relevance of economic outcomes across different payer contexts.^[3]^ Evidence from China and other settings also points to substantial direct medical costs and quality-of-life impacts in HCC, supporting the importance of incorporating financial endpoints into cancer care evaluations.^[4]^

“Financial toxicity” (FT) has emerged as a patient-centered construct capturing the adverse financial effects of cancer and its treatment, encompassing material hardship (e.g., borrowing money, selling assets), psychological distress (financial worry), and coping behaviors that may alter care-seeking. A widely used patient-reported outcome measure is the COmprehensive Score for FT (COST), an 11-item instrument (typically scored 0–44) in which lower scores indicate worse FT.^[5]^ The Functional Assessment of Chronic Illness Therapy organization provides additional instrument context and resources for COST use in clinical and research settings.^[6]^ Importantly, FT is not only a financial metric; it is associated with broader patient outcomes, including health-related quality of life, as summarized in systematic review evidence examining FT measured by COST and related tools.^[7]^

Although COST and related measures are often analyzed as a single continuous total score, FT is inherently multidimensional. Patients with similar overall FT scores may differ markedly in the balance between objective hardship and subjective distress, or in the extent and type of coping behaviors. Such heterogeneity has practical implications: different FT patterns may require different interventions (e.g., financial navigation, social assistance linkage, insurance optimization, or care coordination). Person-centered analytic approaches such as latent profile analysis (LPA) offer a principled way to identify unobserved subgroups that share similar FT patterns across multiple indicators, producing clinically interpretable profiles that can support targeted screening and tailored support. Methodologically, when relating latent profiles to external outcomes (e.g., utilization or spending), bias-adjusted 3-step approaches have been recommended to account for classification uncertainty and reduce distortion of profile definitions that may occur when outcomes are included directly in the measurement model.^[8]^

FT may also shape healthcare utilization in complex ways. Affordability concerns can lead some patients to reduce routine outpatient follow-up or forgo recommended monitoring and supportive care, potentially compromising continuity and early management of complications. Conversely, reduced ambulatory engagement may increase reliance on acute services (emergency department (ED) visits or inpatient admissions) when symptoms worsen or treatment complications arise. In PLC, where liver dysfunction and treatment-related adverse events can evolve rapidly, shifts from planned to unplanned care may be particularly consequential for both outcomes and costs. Understanding how distinct FT profiles relate to outpatient care versus acute utilization can therefore inform clinical management (e.g., proactive follow-up for high-risk profiles) and system design (e.g., embedding financial counseling into oncology pathways).

In parallel, FT intersects with catastrophic health expenditure (CHE), a core indicator of financial protection used in monitoring progress toward universal health coverage. Under WHO indicator metadata, catastrophic health spending is commonly defined as OOP payments exceeding 40% of a household’s capacity to pay, where capacity to pay is total household consumption minus a standard amount required to cover basic needs (including food, housing, and utilities).^[9]^ The 40% capacity-to-pay approach is also aligned with foundational cross-country methodology that operationalizes catastrophic spending relative to resources remaining after subsistence needs have been met.^[10]^

Despite increasing attention to FT and CHE in oncology, evidence remains limited on how distinct FT profiles relate jointly to healthcare utilization patterns and CHE risk among PLC patients. Addressing this gap can clarify which subgroups are most vulnerable to cost-related care fragmentation and catastrophic spending, thereby informing targeted interventions and policy measures. Therefore, this study aimed to identify latent profiles of FT among PLC patients using LPA, and evaluate associations between FT profiles, healthcare utilization (outpatient visits, inpatient admissions, ED visits), and CHE defined by the WHO 40% capacity-to-pay threshold.

## 2. Methods

### 2.1. Study design

This hospital-based retrospective cohort study was conducted in an oncology/hepatobiliary care setting in Chifeng, Inner Mongolia Autonomous Region, China. The study was approved by the Ethics Committee of Chifeng University. Consecutive adult patients with PLC who received inpatient and/or outpatient tumor-related care between December 2024 and December 2025 were enrolled. The index date was defined as the date of the patient’s first tumor-related visit during the study period. A 12-month follow-up window was used to assess healthcare utilization and CHE. Eligible patients were aged ≥ 18 years with PLC confirmed by pathology and/or guideline-consistent imaging criteria. Patients were excluded if they had incomplete key exposure data (FT indicators) or missing primary outcomes (healthcare utilization or expenditure information). Informed consent was obtained as required.

### 2.2. FT measures

FT was assessed using the Comprehensive Score for FT (COST; range 0–44), with lower scores indicating worse FT. In addition to the COST score, multidimensional FT indicators were assessed to capture material, psychological, and behavioral dimensions of financial burden. Material hardship was measured as the number of endorsed financial difficulties related to cancer care, such as difficulty paying medical bills, borrowing money, using savings, selling assets, or reducing spending on basic needs. Financial worry was assessed using a 0 to 10 self-rated item, with higher scores indicating greater concern about medical expenses and household financial security. Coping behaviors were coded as yes/no according to whether patients reported at least 1 cost-related coping strategy, such as delaying or reducing care, seeking cheaper services, borrowing money, or adjusting household spending because of treatment costs. All FT indicators were standardized as *z*-scores before LPA.

### 2.3. Healthcare utilization and CHE

Healthcare utilization during the 12-month window included annual counts of outpatient visits, inpatient admissions, and ED visits. CHE was defined as annual household OOP health spending exceeding 40% of household capacity to pay, calculated as total household consumption minus basic-needs expenditure (food, housing, and utilities). Sensitivity analyses applied an alternative threshold of 25% of capacity to pay.

### 2.4. Data collection

Data were extracted from electronic medical records and structured questionnaires using a standardized case report form. Clinical variables, including diagnosis, tumor stage, comorbidity burden, liver function indicators where available, treatment modality, and healthcare utilization, were primarily obtained from electronic medical records. Sociodemographic variables, household economic information, OOP expenditure, household consumption, COST score, material hardship, financial worry, and coping behaviors were obtained from structured questionnaires. Key variables were independently checked by 2 investigators, and discrepancies were resolved by consensus. When expenditure-related information was available from more than 1 source, questionnaire responses were cross-checked against billing or medical-record information to improve accuracy.

### 2.5. Statistical analysis

LPA was conducted to identify latent FT profiles using Mplus (latent variable mixture modeling). Models with 1 to 5 profiles were compared using Akaike information criterion, Bayesian information criterion, adjusted Bayesian information criterion, entropy, and interpretability; the final solution avoided very small classes. To relate FT profiles to distal outcomes while accounting for classification uncertainty, a bias-adjusted 3-step approach (Bolck-Croon-Hagenaars method/3-step concept) was applied. Count outcomes (utilization) were analyzed using negative binomial regression and reported as adjusted incidence rate ratios (aIRR) with 95% confidence intervals. CHE was analyzed using logistic regression and reported as adjusted odds ratios with 95% confidence intervals. Regression analyses were performed in R (for model fitting and inference) and tables/figures were produced in R. All models adjusted for prespecified covariates (age, sex, income/insurance, stage, treatment modality, and comorbidity burden). Two-sided *P* < .05 was considered statistically significant.

## 3. Results

### 3.1. Participant characteristics

A total of 720 patients with PLC were included during the 12-month observation window (December 2024–December 2025). The mean COST score was 25.0 (standard deviation 10.9). Overall, CHE (OOP > 40% capacity to pay) occurred in 27.0% of participants. Mean annual healthcare utilization was 10.0 outpatient visits, 1.25 inpatient admissions, and 0.56 ED visits (Table 1).

**Table 1:** Key characteristics and outcomes by FT profile (Dec 2024–Dec 2025; N = 720).

Variable	Overall	Low FT	Moderate FT	High FT
N (%)	720	324 (45.0)	270 (37.5)	126 (17.5)
COST (0–44), mean (SD)	25.0 (10.9)	31.8 (7.5)	23.0 (7.8)	12.0 (8.2)
Material hardship (0–5), mean (SD)	1.21 (1.23)	0.55 (0.80)	1.35 (1.10)	2.60 (1.30)
Financial worry (0–10), mean (SD)	4.56 (2.35)	3.05 (2.00)	5.10 (2.05)	7.30 (1.70)
Coping behaviors, %	32.8	16.0	38.0	65.0
Outpatient visits/year, mean	10.0	11.3	9.2	8.6
Inpatient admissions/year, mean	1.25	1.02	1.31	1.72
ED visits/year, mean	0.56	0.40	0.58	0.90
CHE (OOP > 40% capacity-to-pay), %	27.0	18.2	28.9	45.2

CHE = catastrophic health expenditure, COST = higher scores indicate lower financial toxicity, ED = emergency department, FT = financial toxicity, N = number of patients, OOP = out-of-pocket, SD = standard deviation.

### 3.2. Latent profiles of FT

LPA indicated that a 3-profile solution provided the best balance of fit, parsimony, and interpretability (entropy = 0.86; Lo-Mendell-Rubin test *P* = .012) (Table 2). The profiles demonstrated a monotonic gradient of worsening FT across COST, material hardship, financial worry, and coping behaviors. Low FT accounted for 45.0% (n = 324), moderate FT for 37.5% (n = 270), and high FT for 17.5% (n = 126) (Table 1).

**Table 2:** Fit indices for latent profile models.

# Profiles	AIC	BIC	aBIC	Entropy	LMR *P* value
1	7980	8040	8000	-	-
2	7425	7515	7455	0.79	< .001
3	7210	7330	7250	0.86	.012
4	7178	7328	7228	0.87	.192
5	7160	7340	7220	0.88	.413

The final model was selected based on information criteria, entropy, LMR test, and interpretability; solutions with very small classes were avoided.

AIC = Akaike information criterion, aBIC = adjusted Bayesian information criterion, BIC = Bayesian information criterion, LMR = Lo-Mendell-Rubin test.

### 3.3. Associations with healthcare utilization

In covariate-adjusted negative binomial models, higher FT profiles were associated with a shift from routine ambulatory care toward acute care utilization (Table 3). Compared with low FT, moderate and high FT had lower outpatient visit rates (aIRR 0.83 and 0.78, respectively), but higher inpatient admission rates (aIRR 1.28 and 1.62) and ED visit rates (aIRR 1.39 and 1.85), with all associations statistically significant.

**Table 3:** Associations between FT profiles and healthcare utilization (negative binomial regression).

Outcome	Moderate vs Low aIRR (95% CI)	*P* value	High vs Low aIRR (95% CI)	*P* value
Outpatient visits	0.83 (0.76–0.91)	< .001	0.78 (0.69–0.88)	< .001
Inpatient admissions	1.28 (1.10–1.50)	.002	1.62 (1.33–1.97)	< .001
ED visits	1.39 (1.11–1.74)	.004	1.85 (1.40–2.43)	< .001

Model adjusted for prespecified sociodemographic and clinical covariates (age, sex, income/insurance, stage, treatment modality, comorbidity burden).

aIRR = adjusted incidence rate ratio, CI = confidence interval, ED = emergency department, FT = financial toxicity.

### 3.4. Associations with CHE

CHE prevalence increased stepwise across FT profiles (18.2%, 28.9%, and 45.2% for low, moderate, and high FT, respectively) (Table 1). In adjusted logistic regression, moderate FT and high FT were associated with significantly higher odds of CHE than low FT (adjusted odds ratio 1.86 and 3.65, respectively) (Table 4). Findings were robust in sensitivity analysis using the 25% capacity-to-pay threshold (Table 4).

**Table 4:** Associations between FT profiles and catastrophic health expenditure (logistic regression).

CHE definition	Moderate vs Low aOR (95% CI)	*P* value	High vs Low aOR (95% CI)	*P* value
Primary: OOP > 40% capacity-to-pay	1.86 (1.30–2.67)	< .001	3.65 (2.41–5.52)	< .001
Sensitivity: OOP > 25% capacity-to-pay	1.71 (1.24–2.37)	.001	3.18 (2.19–4.61)	< .001

Model adjusted for prespecified sociodemographic and clinical covariates (age, sex, income/insurance, stage, treatment modality, comorbidity burden).

aOR = adjusted odds ratio, CHE = catastrophic health expenditure, CI = confidence interval, FT = financial toxicity, OOP = out-of-pocket.

## 4. Discussion

Our findings add to the growing evidence that the burden of PLC extends beyond clinical outcomes and includes substantial financial consequences for patients and households. In a 12-month cohort, we identified 3 distinct FT profiles and observed a consistent gradient in both healthcare utilization patterns and CHE risk. This aligns with the broader global context in which liver cancer remains a high-mortality malignancy, and survivorship increasingly requires attention to patient-centered and system outcomes rather than survival alone.^[11]^ Within PLC specifically, emerging studies have documented meaningful OOP expenses and financial strain among patients with HCC. For example, United States-based data show that a sizable proportion of patients with HCC experience high financial burden and cost-related distress, highlighting the relevance of FT as a practical dimension of hepatology-oncology care.^[12]^ Medicare analyses further demonstrate that the first year after HCC diagnosis is associated with substantial costs and patient liabilities, supporting the plausibility that financial stress may accumulate quickly during diagnosis, treatment initiation, and early follow-up.^[13]^ Evidence from China and other Asian settings similarly indicates a considerable economic burden alongside quality-of-life impairment in HCC, suggesting that FT is not limited to any single financing system and may be particularly salient where advanced disease and multimodal therapies are common.^[14]^

A key contribution of this study is the use of a person-centered approach to characterize heterogeneity in FT. The LPA provides an interpretable classification of patients into low, moderate, and high FT profiles characterized by coherent patterns across COST, hardship indicators, and coping behaviors. This typology may support targeted screening and stratified supportive care pathways that better match the needs of patients with differing combinations of material hardship and financial distress.^[15–18]^

The observed associations between FT profiles and healthcare utilization suggest a clinically important “care pattern shift” linked to affordability stress. Compared with low FT, moderate and high FT profiles were associated with fewer outpatient visits but more inpatient admissions and ED visits. One plausible interpretation is that financial strain reduces engagement with planned ambulatory care (such as surveillance, supportive care visits, or timely management of adverse events) while increasing reliance on unplanned acute care when symptoms worsen or complications arise. Such pathways are especially plausible in PLC, where hepatic decompensation and treatment toxicities can evolve rapidly, making missed follow-up or delayed management potentially consequential. While causality cannot be confirmed, the consistency of the pattern after covariate adjustment suggests that FT profiles may identify patients at risk of cost-related care fragmentation. Methodologically, linking LPA-derived profiles to distal outcomes raises concerns about classification uncertainty; adopting bias-adjusted 3-step strategies has been recommended to preserve profile definitions while enabling valid inference about external outcomes.^[19]^ The strength and monotonicity of associations we observed (particularly for acute care utilization) support the clinical interpretability of the selected profile solution.

The CHE results underscore that FT is not merely subjective distress; it is closely tied to objective financial protection outcomes. CHE prevalence increased stepwise from low to high FT, and adjusted odds of CHE were substantially higher in moderate and high FT profiles. These findings are consistent with the conceptual model that cancer-related costs can generate both immediate OOP payments and longer-term coping behaviors (debt, asset depletion, or delayed care), which together elevate the probability that household health spending exceeds the threshold deemed catastrophic. Seminal work in oncology has shown that patients experiencing financial burden may alter care, including delaying or forgoing recommended services, which can create downstream clinical deterioration and potentially higher-cost acute events.^[20]^ Our utilization findings mirror this pattern at the health-system level: reduced outpatient engagement alongside increased inpatient and ED use is compatible with cost-related underuse of planned care followed by compensatory acute care. Additionally, clinician-patient communication about costs may be an actionable leverage point. Survey evidence indicates that many patients want cost conversations, yet relatively few have them; among those who do, a substantial proportion report lowered costs through assistance programs, insurance appeals, or adjustments to testing or medications.^[21]^ For PLC care pathways (often involving repeated imaging, procedures, and expensive systemic therapies) normalizing cost discussions and integrating financial counseling could plausibly reduce avoidable financial harm while supporting adherence and continuity.

These findings have several implications for practice and policy. First, incorporating brief FT screening (e.g., COST plus targeted hardship questions) into routine PLC care could help identify high-risk patients early, before financial stress translates into care disruption. Reviews of FT emphasize that interventions may need to operate at multiple levels, including patient navigation, transparent pricing information, and supportive insurance design, rather than relying solely on individual coping strategies.^[22]^ Second, our results suggest that FT profiling could be used to prioritize proactive outpatient follow-up, symptom monitoring, and supportive care for high-risk groups, with the aim of reducing unplanned acute care. Third, the CHE gradient across FT profiles highlights the importance of financial protection mechanisms. Under WHO indicator metadata, CHE is commonly defined as OOP spending exceeding 40% of household capacity to pay (after basic needs), making it a standardized benchmark for monitoring financial risk protection and universal health coverage progress.^[23]^ The foundational multicountry methodology proposed by Xu and colleagues provides the rationale for this approach and underscores that catastrophic spending reflects household resources remaining after subsistence needs are met.^[24]^ By demonstrating that FT profiles strongly stratify CHE risk, our findings support using FT screening as a practical, patient-centered complement to system-level CHE monitoring, potentially enabling earlier mitigation before households cross catastrophic thresholds. More broadly, WHO frameworks emphasize financial protection as a core dimension of universal health coverage; reducing OOP-driven hardship remains a central health-system goal, especially for high-cost chronic conditions such as cancer.^[25]^

Several limitations merit consideration. First, as an observational cohort analysis, unmeasured confounding and reverse causality remain possible. Second, CHE estimation depends on accurate reporting or capture of OOP spending and household consumption. Third, generalizability may be limited by the care setting and financing context; replication across regions and insurance structures is warranted. Future research should evaluate whether FT-targeted interventions, such as structured financial navigation, standardized cost communication prompts, and linkage to assistance programs, can reduce acute care reliance and CHE incidence in PLC populations.

### 4.1. Conclusion

In conclusion, we identified 3 clinically interpretable FT profiles among PLC patients and found that worsening FT was associated with a shift toward higher acute care utilization and markedly higher CHE risk. These findings support routine FT screening and targeted, profile-informed interventions as potential strategies to improve care continuity and strengthen financial protection in PLC management.

## Author contributions

**Conceptualization:** Hongwei Li, Lihong Yang, Li Zhao, Wan Wang, Hanxue Mei.

**Data curation:** Hongwei Li, Lihong Yang, Li Zhao, Wan Wang, Hanxue Mei.

**Formal analysis:** Hongwei Li, Lihong Yang, Li Zhao, Wan Wang, Hanxue Mei.

**Funding acquisition:** Hongwei Li, Lihong Yang.

**Investigation:** Hongwei Li.

**Writing** – **original draft:** Hongwei Li.

**Writing** – **review & editing:** Hongwei Li.
